# Ethanol Separation from an Ethanol–Water Solution Using Vacuum Membrane Distillation

**DOI:** 10.3390/membranes12080807

**Published:** 2022-08-20

**Authors:** Abeer G. Nassif, Salah S. Ibrahim, Hasan Sh. Majdi, Qusay F. Alsalhy

**Affiliations:** 1 Membrane Technology Research Unit, Department of Chemical Engineering, University of Technology-Iraq, Alsinaa Street, Baghdad 10066, Iraq; 2 Department of Chemical Engineering and Petroleum Industries, Al-Mustaqbal University College, Babylon 51001, Iraq

**Keywords:** vacuum membrane distillation, PTEF membrane, separation ethanol-water solution, optimization

## Abstract

The vacuum membrane distillation (VMD) process was applied to separate ethanol from a simulated ethanol–water solution using a commercial polytetrafluoroethylene (PTFE) membrane. The presence of ethanol in the ethanol–water solution with a 2 wt.% ethanol concentration at a temperature above 40 °C during the MD process may result in membrane failure due to an increase in the chance of the PTFE membrane wetting at high temperatures. Therefore, the operating temperature in this study was not higher than 35 °C, with an initial ethanol concentration up to 10 wt.%. This work focuses on optimizing the VMD operating parameters using the Taguchi technique based on an analysis of variance (ANOVA). It was found that the feed temperature was the most-affected parameter, leading to a significant increase in the permeation flux of the PTFE membrane. Our results also showed that the permeate flux was reported at about 24.145 kg/m^2^·h, with a separation factor of 8.6 of the permeate under the operating conditions of 2 wt.%, 30 °C, 60 mm Hg(abs), and 0.6 L/min feed (concentration, temperature, permeate vacuum pressure, and flow rate, respectively). The initial feed concentration, vacuum pressure, and feed flow rate have a lower impact on the permeation flux.

## 1. Introduction

Ethanol is the most-utilized biofuel worldwide, and its utility is getting to be more far-reaching, with prospects of a growing population and thus utilization around the world. Recently, after the coronavirus pandemic, the use of ethanol has become more widespread [1]. In spite of all the benefits, the use of ethanol also raises concerns about the intemperate utilization of water and pollution, among other environmental impacts. The growth of biofuel production (especially absolute ethanol) has stimulated the search for new technologies that can enable these technologies to be recycled and enriched more efficiently and profitably. While ethanol production has many advantages, it is also a source of environmental concern because of the high demand for water and the resulting pollutants. One of the most pressing issues is to find ways to reduce production costs while still addressing environmental concerns [2]. Process intensification may be a way to improve the process industry’s competitiveness by increasing the efficiency, productivity, and speed of industrial processes while simultaneously reducing their energy consumption and waste generation [3]. To enhance the efficiency of the fermentation process, a variety of ethanol-extraction techniques have been employed. The increased production of biofuels (particularly anhydrous ethanol) has prompted the quest for novel techniques that allow for a more efficient and lucrative recovery and concentration of these products; membrane technology is one of them [1]. There are two specific membrane separation processes that can be used to recover, remove, or separate alcohols; the common one is the pervaporation process while the second is membrane distillation.

Membrane distillation (MD) is one of the separation methods that can be applied to single-component separation (e.g., water treatment, desalination) [4], binary mixture separations (e.g., the concentration of dilute ethanol solutions), and separation of volatile compounds from multi-component mixtures (e.g., recovery of flavors from aqueous solutions) [4]. There are four standard MD configurations: direct contact membrane distillation (DCMD), vacuum membrane distillation (VMD), air gap membrane distillation (AGMD), and sweeping gas membrane distillation (SGMD) [2]. As one of the most promising separation technologies, MD was used for desalination, water purification, separation of the volatile component, the concentration of non-volatile solutions, wastewater treatment, and azeotropic mixture separation [4]. MD for recovering alcohols is a technique for separating a liquid mixture utilizing porous membranes, with a partial pressure gradient across the membrane as the driving force. To flow through the membrane, the volatile components must change phase throughout the process [5]. The main advantage of this technology is the ability to continually remove ethanol throughout the fuel alcohol synthesis stage, avoiding fermentation by-product inhibition and enhancing production [6]. 

Membrane processes are also regarded as clean, simple to run, and linear scaling processes, which are key characteristics when considering industrial applications. Pervaporation and MD are two membrane techniques that have been researched for ethanol separation. The fundamental distinction between these two methods is the kind of membranes used where pervaporation uses non-porous (dense) membranes, whereas MD employs porous membranes. This membrane characteristic enables MD to extract ethanol from ethanol–water mixtures more quickly (almost 100 times quicker) [4]. 

Among the four setups in MD, VMD and SGMD draw more attention as ethanol separation methods, since both allow for a higher flux of the permeate with a decent concentration of alcohol [6,7,8,9]. From the literature, it was found that only a few studies have been carried out on the MD process applied to recover alcohols (mostly ethanol). Studies have mainly focused on the experimental analysis, including theoretical analysis (simulation model) and the impact of the operating conditions on each of them [10]. Benavides-Prada et al. [4] studied the VMD technique through a simplified mathematical model, based on the energy and mass transfer across the membrane for diluted mixtures of ethanol–water. The simplification of the model was done through validation of the hypothesis considered for this application. A finite difference method was used to solve the mathematical model applying the software tool MATLAB^®^ ver.6.2 (MathWorks, Natick, MA, USA), with the data reported in the literature used for validation of the model. Gryta et al. [11] investigated a range of ethanol concentrations in a fermentation medium and concluded that the selectivity depends on the feed temperature; indeed, increasing from 30 °C to 60 °C, the enrichment factor varied from 4 to 6. Cotamo-De La Espriella et al. [7] analyzed and compared two configurations, VMD and SGMD, using prepared water–ethanol solutions and water–ethanol mixtures from fermented broths. At first, they considered working under moderate vacuum conditions for VMD, using membranes with a pore diameter up to 5 µm for SGMD; this was the first study reporting the comparison of the two techniques under similar operating conditions. Izquierdo-Gil and Jonsson [10] investigated the influence of concentration polarization on flux and ethanol selectivity using VMD and obtained a separation factor in the range of 5.2–7.9.

However, ethanol is considered a volatile organic component, and therefore it could be a wetting agent for hydrophobic membranes. Thus, a challenge is the use of conventional hydrophobic membranes for treating ethanol–water mixtures using MD. In addition, membrane wetting is a particularly important challenge for VMD because the pressure difference between the liquid and membrane pores may near the liquid entry pressure of traditional membranes of MD. Hence, for successful used of VMD in the removal of ethanol from aqueous solutions, membrane wetting should be as far off as possible. 

It is essential to mention here that the presence of ethanol in an ethanol–water solution with a 2 wt.% ethanol concentration at a temperature above 40 °C during the MD process may result in membrane failure due to an increase in the chance of PTFE membrane wetting at high temperatures. Some of the research works found in the literature used operating temperatures higher than 40 °C for the PTFE membrane [9,10,11,12,13,14]. Therefore, the operating temperatures in this study were between 20 and 35 °C, with an ethanol initial concentration up to 10 wt.%. Moreover, in this study, a VMD system was built in order to perform the separation of ethanol from its aqueous solution. In the current study, the effect of the operating conditions, such as the feed temperature (20 to 35 °C), initial ethanol concentration (2–10 wt.%), flow rate (0.3–0.6 L/min), and vacuum pressure (60–210 mmHg (abs)), on the permeation flux and separation factor was investigated. A commercial polytetrafluoroethylene (PTFE) flat sheet membrane was used in the VMD process. Furthermore, by using a statistical analysis method (ANOVA), the parameter sequences that have a more substantial impact on the permeate flux were investigated, as well as the optimum operating settings were detected. A comparison of the efficiency of VMD in the current work with the efficiency of VMD found in the literature using different types of the membranes was also performed. 

## 2. Materials and Methods

### 2.1. Materials

**Figure 1:**
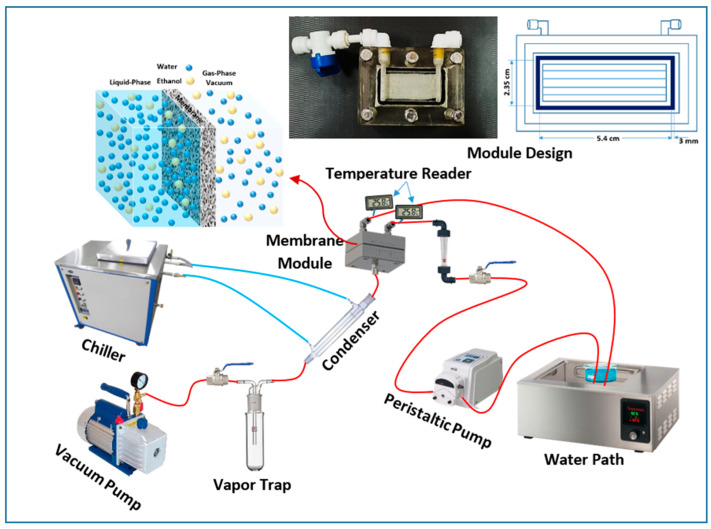
Schematic diagram of the vacuum membrane distillation setup and module design.

A rectangle plate-and-frame membrane module was designed and made of acrylic polymer material (transparent plastic) to resist corrosion and dissolving by the ethanol solution and to have high heat-transfer resistance. This module has been used in the VMD process, which has three closets, two for the hot ethanol/water solution (input and output) and the other for the permeate vapor withdrawal (outlet), as shown in Figure 1. This module was carried out using a computer numeric control machine (CNC) and CAD-CAM program according to the dimensions shown in Figure 1. A commercial flat-sheet polytetrafluoroethylene (PTFE) membrane (A Sterlitech^®^ Sepa CF module (Sterlitech Corporation, Auburn, WA, USA)) was used. The characteristics of this flat-sheet membrane are summarized in Table 1. The outer area of the module is about 54 cm^2^ and the effective area is about 12.7 cm^2^. The polymeric PTFE membrane was inserted in the middle of the module, which separates the feed and permeate chambers. The membrane was supported by plastic mesh. The hot feed solution flowed through the feed chamber side while a vacuum was applied to the other side (permeate chamber). The cold distillate water was circulated through the condenser to condensate the permeate flux vapor that passed through.

### 2.2. Feed Solution

The simulated feed solution (ethanol–water) was prepared at various concentrations (2, 5, and 10 wt.%) of ethanol using pure ethanol (99.9%) (Scharlab S.L., Barcelona, Spain). Each sample was mixed in a conical flask containing 400 mL of distilled water and then mixed with a certain weighted amount of ethanol to obtain the required concentration of the simulated feed. A calibrated portable refractometer’s measurement (Balance World Inc., Model ATC-Refractometer, China) was used to measure the concertation of ethanol alcohol. The penetration of the ethanol across the membrane was detected by measuring its concentration in the aqueous solution collected in the vapor glass trap. This measured concentration was used to estimate the separation factor according to Equation (1) [12].

(1) $$\alpha =\frac{y_{e,p}/y_{w,p}}{x_{e,f}/x_{w,f}}$$

where α is the separation factor or membrane selectivity, $y_{e,p}$ is the mole fraction of ethanol in the permeate, $y_{w,p}$ is the mole fraction of water in the permeate, $x_{e,f}$ is the mole fraction of ethanol in the bulk liquid feed, and $x_{w,f}$ is the mole fraction of water in the feed bulk liquid.

### 2.3. Experimental Setup

Figure 1 shows a schematic diagram of the VMD process that was utilized in this study. Heat losses were minimized by insulating all of the pipelines and feed reservoirs. A water bath with a temperature controller was used to monitor and control the feed temperatures at the module’s input. The feed liquid of about 500 mL in the reservoir was heated to the desired temperature before being continuously pumped to the membrane module via a rotameter. The permeate side apparatus included a condenser and a vacuum pump. The vapor could be quickly condensed in a glass condenser jacket and was weighed at a certain time by an electronic balance. The feed flowed back to the reservoir to be reheated again to the required feed temperature after being recirculated again to the module. The samples were also returned to the reservoir after testing to preserve feed concentration stability. For cooling requirements, ice was employed as the coolant in a cooling water condenser and also for cooling the glass vapor trap to collect and condensate the whole permeate vapor flux. The permeate flux was calculated using Equation (2) below [8,17].

(2) $$J=\frac{V\;\times \rho }{A\times t}$$

where J is the permeate flux (kg/m^2^·h), V is the accumulated volume of fresh water (L), ρ is the water density (kg/L), t is the operational time (hour), and A is the effective membrane area, which can be simply calculated as follows [8,18]:

(3) A=W×L

where W and L are the effective width and length of the membrane (m), respectively.

### 2.4. Characterization Methods

**Figure 2:**
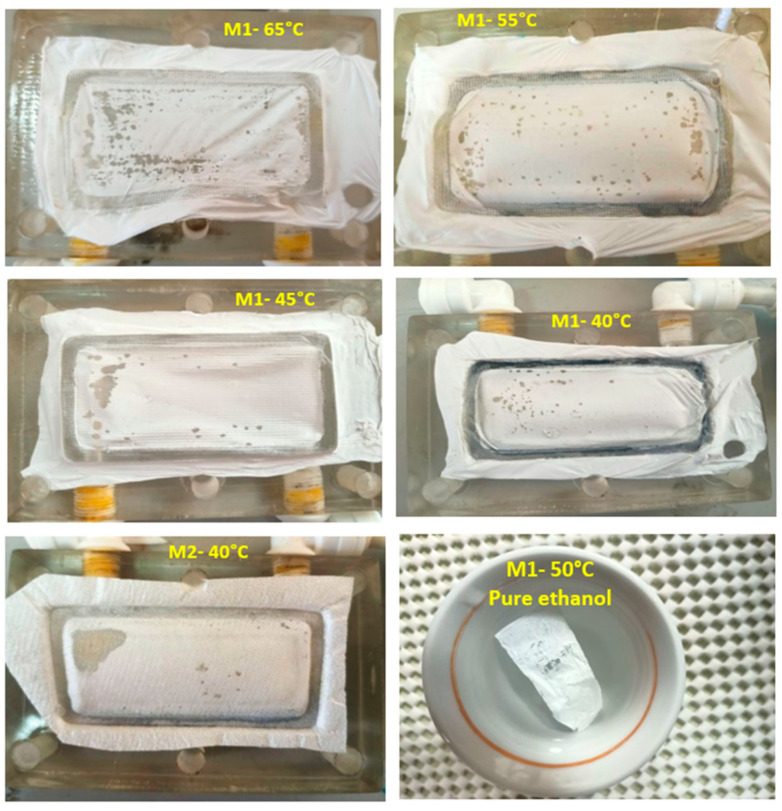
Pictures of the failure of the two types of PTFE membranes (M1 and M2) for a 2 wt.% initial ethanol concentration at 40–65 °C feed temperature, a 0.6 L/min feed flow rate, and 79.9 mbar permeate vacuum pressure.

Experiments were performed in order to evaluate the performance of the VMD process and to investigate the role of the main process variables on the permeate flux and separation factor. For the ethanol–water solution, the influence of temperature, composition, and recirculation rate on the liquid feed side and of the vacuum pressure on the permeate side was studied. Temperature, initial concentration, and recirculation rate were varied in the range of 20 to 35 °C, 2 to 10 wt.%, and 0.3 to 0.6 L/min, respectively. The operating temperature range in this study was because the PTFE membranes could fail due to wetting at higher operating temperatures, even for a lower initial ethanol concentration (2 wt.%), as shown in Figure 2. The downstream pressure range examined strictly depends on the mixture (ethanol–water) under investigation: the value of the downstream pressure must be kept below the equilibrium vapor pressure of the feed, and thus very different pressure ranges can be used for the VMD process.

Practically, the membrane was tested using distilled water without any problem with temperature up to 65 °C, with different values of the feed flow rate and permeate vacuum pressure. A problem appeared especially at a high temperature (more than 40 °C), even when the ethanol presence was at a low concentration, such as in the present work at 2 wt.%. The expected reason may be that, at a high temperature with the presence of ethanol, the chance for membrane wetting is increased due to the significant decay in solution surface tension, as will be clarified in the next sections, leading to membrane failure. It was also found that some small holes appeared on the membrane surface and this indicated that the polymer may be dissolved at these conditions. This result was confirmed when putting a small piece of membrane in pure ethanol at about 50 °C for about 30 min; the membrane wetting was clear, as shown in Figure 2.

To confirm the effect of ethanol presence in the feed on the PTFE membrane, an experiment with operating conditions of a 2 wt.% initial ethanol concentration in the feed, 40 °C feed temperature, a 0.6 L/min feed flow rate, and 79.9 mbar permeate vacuum pressure was repeated using another commercial PTFE membrane (M2) (Chmlab Group 08205 Barcelona, Spain). The new membrane (M2) is different than that used in the present work (M1), and its characteristics are given in Table 1, which had been examined by using it in our previous work [8,15,16] for water desalination using DCMD and SGMD. This new membrane also failed due to wetting at 40 °C, as shown in Figure 2. 

### 2.5. Experimental Design Using the Aguchi Method

The Taguchi technique is frequently used in engineering analysis to arrange experiments to obtain data in a controlled manner and thereby obtain knowledge about a particular process’ behavior. The major benefit of this strategy is its efficacy in terms of the amount of time, effort, and money necessary to run trials, as well as its speed in identifying key components [19]. The degrees of freedom (DOF) of the process parameters, as well as the number of levels of various parameters, influence the choice of a suitable orthogonal array (OA) [19,20]. The DOF can be calculated as follows:

(4) DOF=P(L−1)

where P is the number of parameters and L is the number of levels. Here, four parameters each at three levels would be selected; thus, DOF = 4 (3 − 1) = 8.

The degree of freedom of the OA should, in general, be more than or equal to the process parameters. The standard L_9_(3^4^) orthogonal array has four parameters and three-level columns with eight degrees of freedom. As a consequence, an L_9_(3^4^) orthogonal array with four columns and nine rows was used in this study. Nine experiments were carried out at different parameters using the Taguchi approach. The nine rows correspond to the number of trials and the four columns represent the investigated parameters at three levels for each parameter. The ANOVA statistical technique was used to analyze the experimental data and assess the influence of each component on performance.

## 3. Results and Discussion

### 3.1. Effect of Feed Temperature

**Figure 3:**
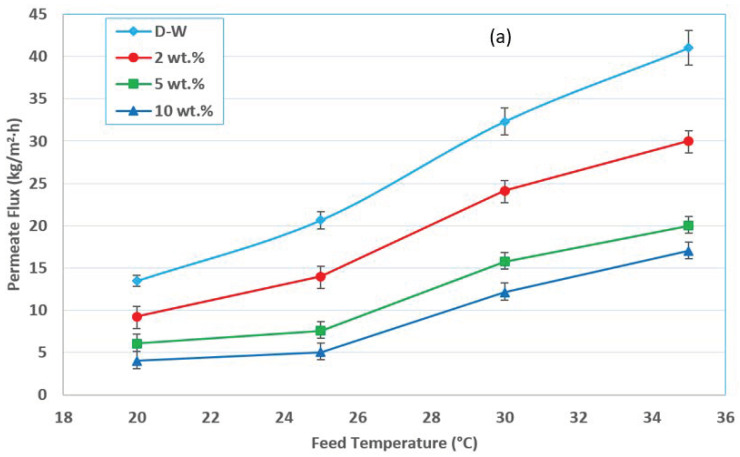

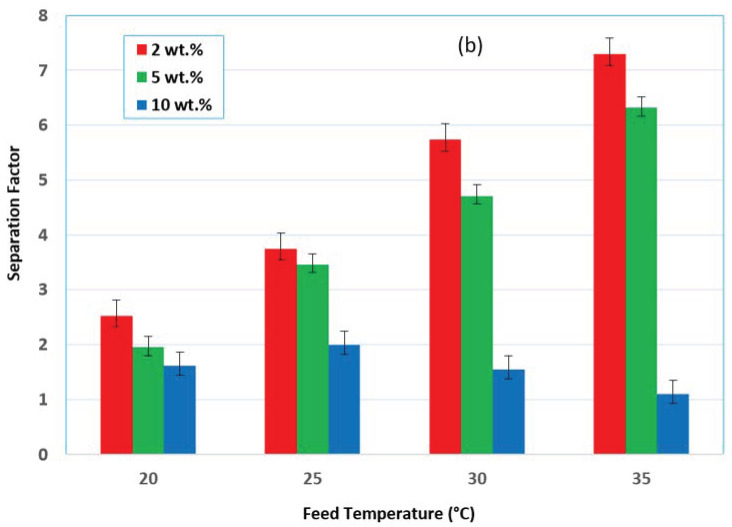

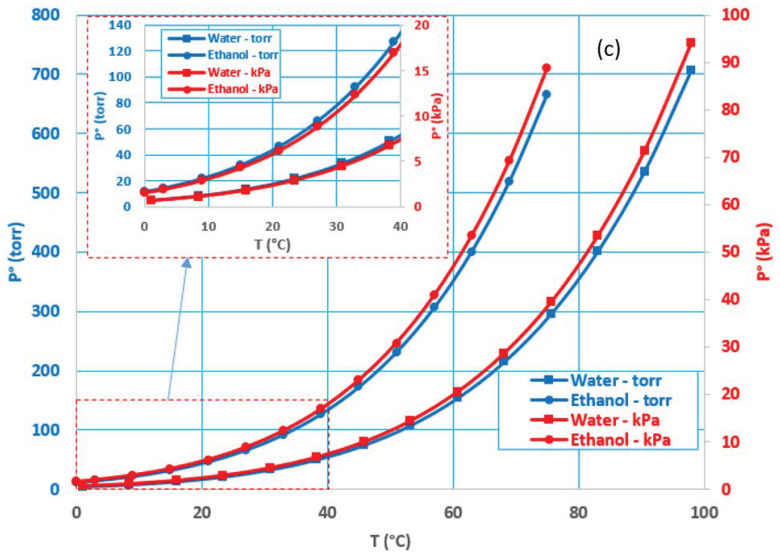

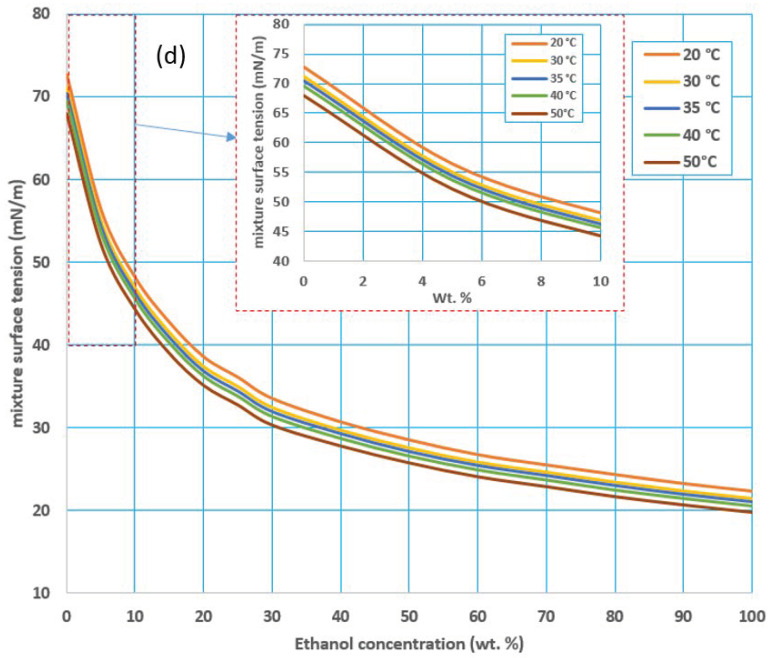
Effect of the feed temperature on (**a**) permeate flux, and (**b**) the separation factor at different concentrations, a 0.6 L/min feed flow rate, and 60 mmHg (abs) pressure. (**c**) Vapor pressure with temperature for water and ethanol. (**d**) The ethanol–water mixture surface tension with temperature.

In the MD process, the feed temperature plays a vital role in terms of its effect on the vapor pressure and the heat transfer within the membrane module. The present study used the VMD process with a PTFE membrane to treat a water–ethanol solution. The fluxes in the water–ethanol solution were studied at different feed temperatures, concentrations, at a constant feed flow rate of 0.6 L/min, and at a vacuum pressure of 60 mmHg (abs), as shown in Figure 3a. In the VMD process, the effect of the feed temperature on the permeate flux is similar to other MD processes in that increasing the feed temperature leads to increasing the permeate flux. The feed temperature is a very sensitive operating parameter, which significantly affects both the permeate flux and the total energy requirement [17,18]. Figure 3a,b shows the effect of feed temperature on the permeation flux and the separation factor at various feed concentrations. It can be seen that with increasing the feed temperature, the permeation flux was increased due to the increase in vapor pressure of the ethanol and water. This phenomenon is explained by the Antoine equation, which connects the equilibrium vapor pressure to the temperature. The exponential behavior of the permeate flux with the operating temperatures is evident, as shown in Figure 3a. It obeys Antoine’s equation, which anticipated an exponential connection between the feed temperature and vapor pressure, and this agrees with the results reported in many studies [9,18]. 

Furthermore, raising the feed temperature improves the increasing rate of the ethanol vapor pressure more than that of water, as shown in Figure 3c. As a result, the ethanol rate of evaporation would be more than that of water, resulting in an increased ethanol flux rather than water at higher temperatures. The separation factor increased as the temperature and concentration were increased, except for the feed of the 10 wt.% ethanol concentration at 30–35 °C, where the separation factors decreased. This is because the non-uniform diameter of the pores at the membrane surface (pore size distribution) makes the membrane susceptible to pore wetting via capillary forces [18,21].

The primary force against pore wetting is the surface tension of the process liquids. Surface tension is a measure of the intermolecular forces present at the surface of a liquid. In other words, it is the amount of force that is required to break the surface of a liquid. A liquid, such as water, with hydrogen bonding present, will have a high surface tension. Because hydrogen bonds are so strong, there will be a higher amount of force needed to break the bonds and distort the surface barrier of the liquid. On the other hand, liquids with weaker intermolecular forces, named surface-active agents (SAA), such as ethanol, correspondingly have a low surface tension. These types of liquids contain bonds that can be broken more easily, so the surface is more likely to be distorted. The surface tension of the liquids generally decreases with an increase in temperature and becomes zero at critical temperatures (when the meniscus between the liquid and the vapor disappears). The decrease in surface tension with an increase in temperature is due to the fact that with the increase in temperature, the kinetic energy of the molecules increases and hence intermolecular attraction decreases. For example, the most significant surface tension value in such systems is for pure water (72.1 mN/m at 25 °C) [21], which is high enough to retard pore wetting via capillary forces. The substantial difference in surface tension between water and ethanol (ethanol surface tension = 22.07 mN/m at 25 °C) [21] causes a dramatic fall in surface tension for ethanol–water mixtures. For low ethanol percentage mixtures, as measured experimentally by Shirazi et al. (2015) [21], it was found that the surface tension decreased by 31.5 percent as the ethanol weight percent increased from 0 to 10%. The mixture surface tension of the ethanol–water solution, as a function of the concentration and temperature, is shown in Figure 3d (the data quoted from Vazquez et al. (1995) [22]), which gives the extent of the decay in the mixture surface tension with respect to ethanol concentrations and temperatures for the present work range. Moreover, this figure clearly points out that the effect of ethanol concentration on the mixture surface tension and thus membrane wetting has a far more significant influence than the temperature effect on the mixture surface tension. The largest pores on the membrane surface begin to wet due to capillary forces at this point. Moreover, the surface tension of the ethanol–water mixtures, on the other hand, begins to drop as the input temperature rises. Thus, pore wetting is more severe at higher temperatures than at lower ones. When the feed temperature and concentration were 35 °C and 10 wt.%, respectively, the pore wetting significantly impacted the separation factor. Of course, in the case of ethanol–water mixtures, the effect of ethanol concentration on pore wetting has a far more significant influence than the temperature effect [23]. Figure 3b shows that the separation factor increases with feed temperature for the initial ethanol concentrations of 2 and 5 wt.%, while for the 10 wt.% concentration it is slightly increased when the temperature was increased from 20 to 25 °C; then it is drops at a higher temperature. This case could be attributed to the possibility of membrane wetting due to lowering the surface tension to an effected value for a certain flow rate and absolute permeate pressure.

**Figure 4:**
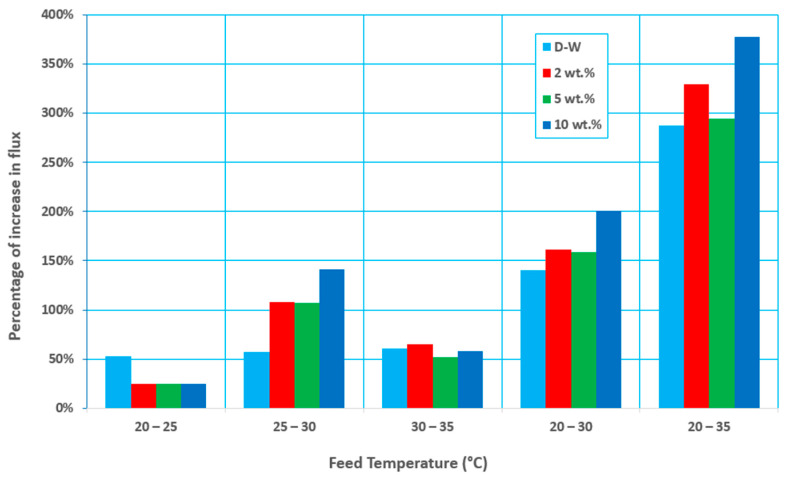
Percentage increase in flux at various temperatures at different concentration factors, a 0.6 L/min feed flow rate, and 60 mmHg (abs) pressure.

Figure 4 demonstrates that the increase in permeation flux for the temperature interval of 25–35 °C is significantly more than for 20–25 °C. This is due to the exponential behavior of the saturated liquid vapor pressure with temperature. As shown in Figure 4, the permeation flux for a solution with a 2 wt.% initial feed concentration increased by about 25% when the feed temperature increased from 20 to 25 °C. However, the permeate flux increased by about 161% when the feed temperature increased from 20 to 30 °C and 329% when the feed temperature increased from 20 to 35 °C.

### 3.2. Effect of Feed Flow Rate

**Figure 5:**
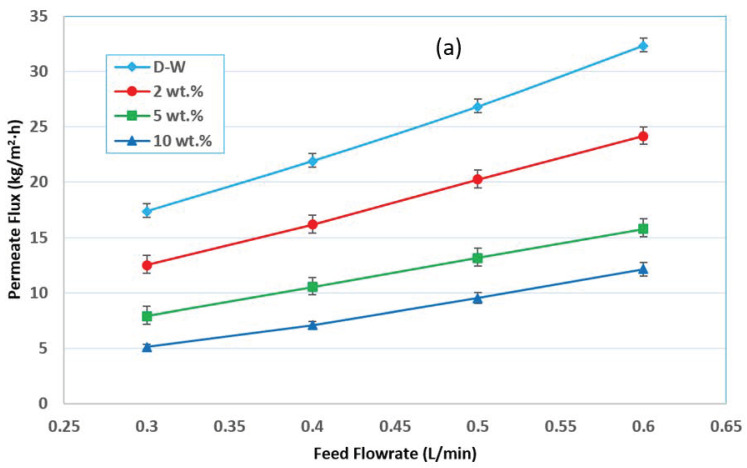

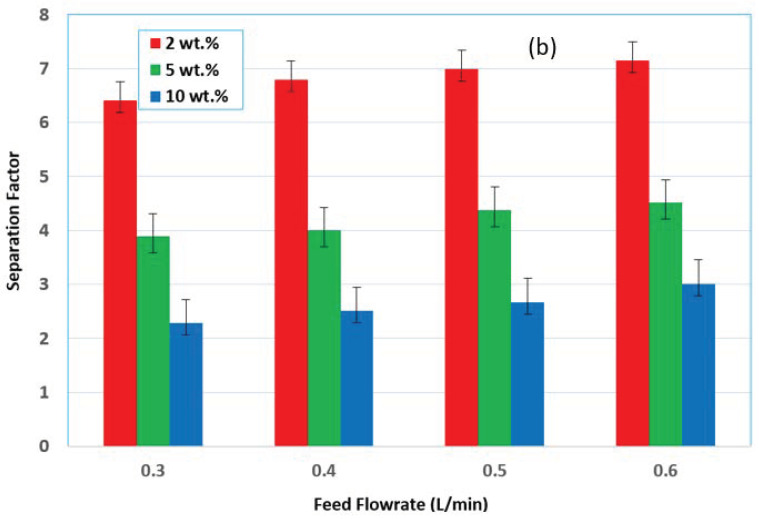
Effect of the feed flow rate on (**a**) the permeate flux, and (**b**) the separation factor at different concentrations of ethanol and at 30 °C and 60 mmHg (abs) pressure.

The effect of feed flow rate on the separation factor and permeate flux was illustrated in Figure 5. It can be seen that both the permeate flux and the separation factor were increased with the increase of the feed flow rate. It could be known that at a higher feed flow rate, the velocity of the feed adjacent to the membrane feed side increased, which increase the Reynolds number and in return convective mass transfer coefficient would be enhanced. Therefore, a thinner mass transfer layer (boundary layer) at the membrane surface could be achieved, and the mass transfer could be enhanced under the condition of a higher feed flow rate. Enhanced mass transfer at the boundary layer at the membrane feed side could reduce the mass transfer resistance and improve the membrane’s separation performance with increasing total flux (higher Ethanol and water flux), as shown in Figure 5. In this case, higher ethanol concentration at permeate side of the membrane would be obtained, with a higher separation factor achieved. The mass transfer resistance of membrane separation included the convective mass transfer resistance through the boundary layer at the membrane feed side and the mass transfer resistance across the membrane. The convective mass transfer resistance was related to the flow rate of the feed (Reynolds number).

In contrast, the mass transfer resistance across the membrane was related to the membrane characteristics (such as membrane porosity, mean pore size, membrane thickness, etc.), independent of the flow rate at the membrane surface. A higher membrane porosity, larger mean pore size, and thinner membrane thickness could promote mass ethanol transfer across the membrane. The water, the convective mass transfer resistance, also could be predominant under a lower flow rate, and the membrane flux could be increased obviously by increasing the feed flow rate. However, the mass transfer resistance across the membrane could be predominant under a higher feed flow rate, and the flux through the membrane could not be increased obviously with the increase in the feed flow rate. Therefore, the permeate flux would be increased identically to the feed flow rate increase, as shown in Figure 5a [12].

**Figure 6:**
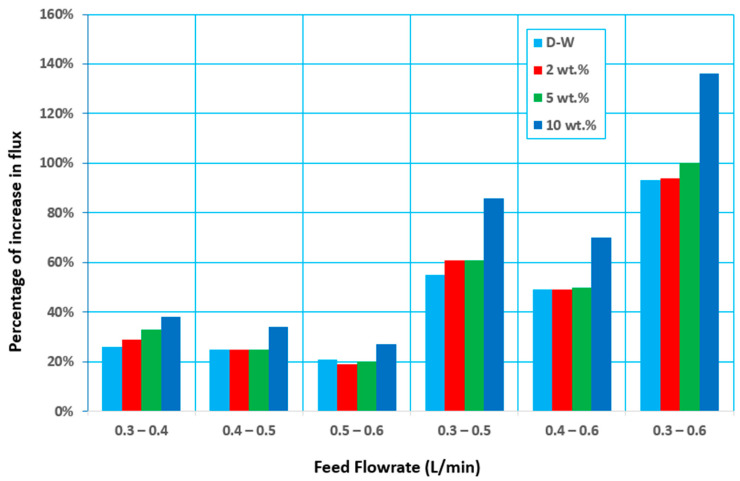
Percentage increase in flux with an increasing feed flow rate at different feed initial concentrations and at 30 °C and 60 mmHg (abs) pressure.

As the feed flow rate increased from 0.3 to 0.4 L/min, the permeate flux increased by about 26–40% and by about 55–142 and 86–246%, respectively, as the feed flow rate increased from 0.3 to 0.5 and 0.6 L/min, as shown in Figure 6. The increase in Reynolds number, which produces greater flow mixing in the channels owing to the turbulence scenario, is responsible for the increase in permeate flux.

### 3.3. Effect of Vacuum Pressure

At any temperature, the equilibrium vapor pressure for the mixture is the bubble point pressure of this mixture (initial boiling). In MD processes, the feed mixture was almost at a pressure above the equilibrium vapor pressure (or temperature below the bubble point temperature of the mixture). However, there are still vapors presence above the liquid surface of pressure equal to the vapor pressure of the mixture. This vapor would be withdrawn by various methods according to the MD configurations. Thus, in VMD, lowering the pressure at the permeate side will provide a higher driving force for convective mass transfer for the vapor to pass across the membrane. In the present study, the vacuum pressure range was 60–210 mmHg (abs) (79.9–279.9 mbar(abs)).

**Figure 7:**
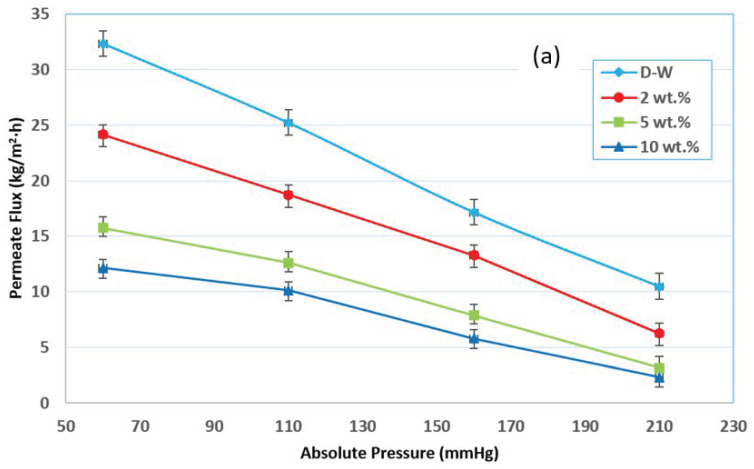

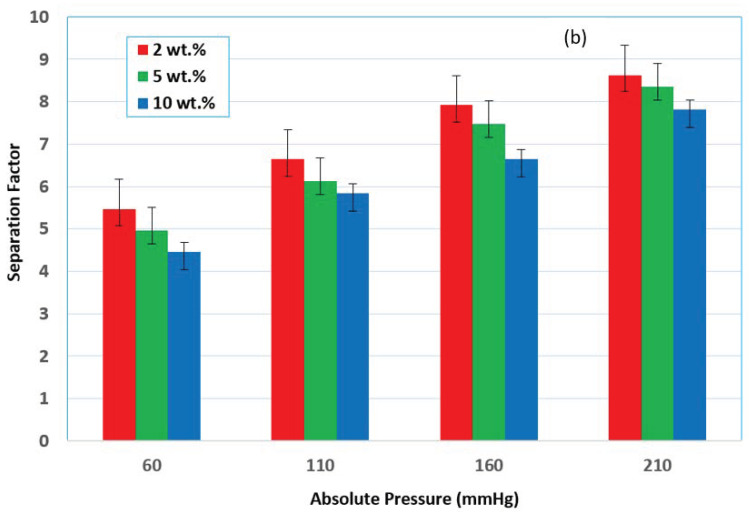
Effect of absolute pressure (in vacuum zone) on (**a**) the permeate flux, and (**b**) the separation factor for a feed temperature at 30 °C, a 0.6 L/min feed flow rate, and different initial concentrations.

In the VMD system, the permeate flux increases as the absolute permeate pressure (in the vacuum zone) decreases. In other words, when the absolute pressure drops, the permeate flux drops across the membrane. Various permeate compositions are produced, depending on the absolute pressure (in the vacuum zone). It showed that when the absolute pressure (in the vacuum zone) at the permeate side is low, the water flux rather than ethanol flux increases, corresponding to the feed temperature of the ethanol–water solution. The same results are shown in other studies [17,24]. The expected reason may be due to the liquid mixture surface tension. The surface tension is inversely proportional with exerted pressure [25]. An increase in pressure increases the surface tension because the inter molecular force of attraction increases. Thus, a decrease in pressure decreases the liquid surface tension and, in return, the wettability chance of the membrane increases. When the absolute permeate pressure (in the vacuum zone) is relatively high, however, in the permeate solution the water concentration reduces, and the ethanol concentration rises. As a result, if a high separation factor is required, the downstream pressure (in the vacuum zone) should be kept high enough to be as far as possible from the membrane wetting. It was noted that the downstream pressure (in the vacuum zone) significantly impacts the volatile solute concentration in the permeate. When the feed’s ethanol concentration was 2 wt.%, the permeate solution could achieve a 7.9 wt.% ethanol concentration in water. These effects may be accomplished by significantly reducing the driving force for permeate water flux while only slightly lowering the downstream absolute pressure, reducing the driving force for ethanol. However, the relative relevance of the polarization effects on the VMD process performance depends on the permeating species and operating variables, such as the downstream pressure, hydrodynamic feed conditions, and the utilized membrane module [12]. Figure 7a illustrates examples of how the total permeate flux increases when the downstream pressure decreases. The water flux is relatively high compared to the ethanol permeate flux at low downstream pressures, and it approaches the total permeate flux. As a result, the concentration of ethanol in the permeate drops dramatically.

**Figure 8:**
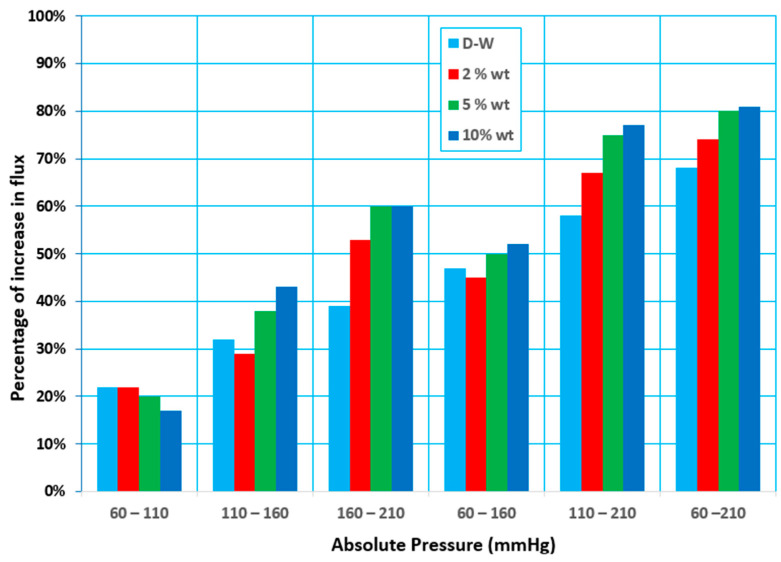
Percentage decrease in permeate flux with increasing absolute pressure at a feed temperature of 30 °C, a 0.6 L/min feed flow rate, and various concentrations.

On the other hand, at higher vacuum pressures, the water flux rapidly declines, and the ethanol concentration (due to its flux) rises above that of water. It is worth noting that lowering the absolute pressure uses more energy while improving the ethanol separation factors, suggesting that the VMD process is better suited to moderate downstream pressures (in the vacuum zone). The separation factor, as shown in Figure 7b, increases as the downstream pressure (in the vacuum zone) increases; this explains the behaviors of mass transfer within the pores of the membrane and the vapor–liquid balance of the ethanol and water. Experimental data were obtained at 30 °C for 5 wt.% ethanol–water mixtures and a 0.6 L/min feed flow rate at 60 to 210 mmHg downstream pressures. The separation factor increases slightly, especially at the higher downstream pressure, which corresponds to higher fluxes; this fact supports the conclusion that the concentration polarization is significant and plays a crucial role in determining the separation efficiency. In the VMD process, the impact of air in the membrane pores on water vapor diffusion through the pores may be ignored, resulting in a reduction in conduction heat transfer across the membrane and an increase in permeate flux [24,26]. Figure 8 shows the proportion of the decrease in permeate flux when the absolute pressure increases.

When the absolute pressure rose from 60 to 110 mmHg (abs), the permeate flux fell from 17 to 22%, and when the absolute pressure climbed from 60 to 160 and 60 to 210 mmHg (abs), the permeate flux reduced from 44 to 52% and 68 to 81%, respectively. The significant increase in the vapor pressure differential (the driving force) between the feed and permeate sides is related to this phenomenon. When an aqueous feed solution contains volatile solutes, both water and the volatile solutes are transported through the membrane and the change in the feed concentration with time allows us to determine the overall mass transfer coefficient (K), which is given by Equation (4). K is influenced by the mean temperature (via mean vapor pressure and vapor viscosity) as well as the membrane structure. $k_{f}$, $k_{m},$ and $k_{p}$ are the mass transfer coefficients of the feed, membrane, and permeate layers, respectively, and are combined by the resistances in the series model. In most studies using VMD, the resistance term corresponding to $k_{m}$ is neglected, mainly when very low downstream pressures are applied [12,21].

(5) $$K=\frac{1}{1/k_{f}+1/k_{m}+1/k_{p}}$$

The saturation pressure, P° (vapor pressure), which can be computed using the Antoine equation, Equation (5) (P° in mmHg and T in °C), is identical to the vapor pressure for the pure water (A = 8.108, B = 1750.286, C = 235) and pure ethanol (A = 8.1122, B = 1592.864, C = 226.184). Because of the low pressure on the permeate side (vacuum), the partial vapor pressure difference between the two sides of the membrane can be expressed using Equation (6). The main factor that should be far away from it in the MD process is the membrane wettability, which, in turn, depended on the liquid entry pressure (LEP) of the membrane. Equation (6) gives the applied partial pressure (∆P_i_) across the membrane for component i. The total pressure difference (∆P = ∑∆P_i_) across the membrane should be less than the LEP of the membrane used to avoid the membrane wetting. In the present study, ∆P was less than the LEP of the membrane used for all experimental conditions.

(6) $${\mathrm{logP}}^{{}^{\circ}}=\left(A-\frac{B}{T+C}\right)$$

(7) $$\Delta P_{i}=x_{i}\alpha_{i}P_{i}^{{}^{\circ}}-y_{i}P_{\mathrm{mp}}$$

where $x_{i}$ and $\alpha_{i}$ are the mole fraction activity coefficients of component i, respectively.

### 3.4. Effect of the Initial Feed Concentration

The studied ethanol concentrations were the initial concentrations of ethanol in the feed mixture, which was about 500 mL for each experimental run. The accumulated amount of the permeation flux, after the end of the experiment (2 h), was little in comparison to feed amount used. Thus, the initial concentration would not be changed to a significant value during the experiment. The use of the MD process for treating a system of volatile organic components–water mixtures is different than that of salt–water systems, since there is no salt in the vapor phase for the desalination system. In the present case, the ethanol and water are transported together across the membrane to the permeate side where the ethanol concentration will be relatively higher than that in the feed according to the separation factor. Thus, the ethanol recovery would be increased by increasing the permeation flux as well as the separation factor for each experimental run; accordingly, for more ethanol recovery, more processing time is required.

**Figure 9:**
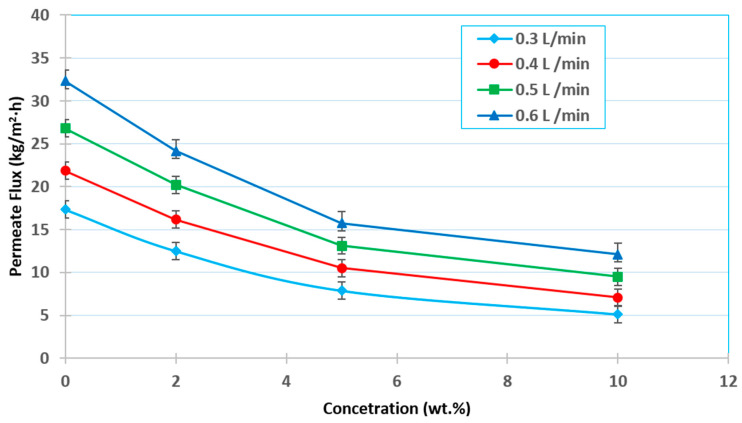
Effect of the initial feed concentration on the permeation flux at a 30 °C feed temperature and 60 mmHg (abs) absolute pressure.

The experiments were carried out at various feed concentrations (i.e., 0, 2, 5, and 10 wt.%) and feed flow rates (i.e., 0.3, 0.4, 0.5, and 0.6 L/min) while both the absolute permeate pressure and feed temperature were constant at 60 mmHg (abs) and 30 °C, respectively, as shown in Figure 9. These results showed that the permeate flux drops when the ethanol content increases. In general, the total permeate flux decreases with an increasing ethanol concentration in the feed due to the chance of membrane wetting, which could increase with a decrease in the liquid mixture surface tension and increasing ethanol concentration, as shown in Figure 3d. Theoretically, the VMD permeate flux should be zero of the nonvolatile component and certainly would exist for the volatile component when the downstream pressure is above the saturation pure water vapor pressure. To avoid wetting the membrane pores, care should be taken when increasing the volatile solute concentration in the feed solution. 

**Figure 10:**
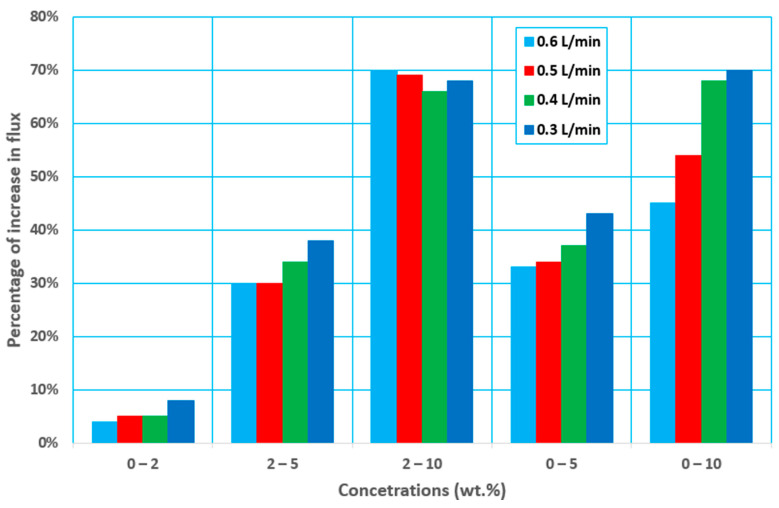
Percentage decrease in permeate flux with an increasing initial ethanol concentration at a feed temperature of 30 °C, 60 mmHg (abs) pressure, and various flow rates.

Figure 10 shows the percentage decrease in permeate flux when the initial ethanol concentration is increased at each feed flow rate. It can be seen that the flux declines rapidly at a low feed flow rate. At 0.3 L/min, the flow declines by around 48–70%, whereas at 0.4 L/min, it drops by about 49–68%. Furthermore, when the flow rate was raised to 0.5 and 0.6 L/min, the drop was around 30–54 percent and 18–45%, respectively. This can be attributed to the influence of the feed flow rate on the Reynolds number, mass transfer coefficient, and heat transfer coefficient, and also the chance of membrane wettability. 

Table 2 depicts the comparison between the current work results and the results found in the literature for ethanol separation using the VMD process in terms of membrane characteristics, operating conditions, permeated flux, and separation factor. As can be seen, the obtained permeation flux in the current work is better than that presented in most of the relevant works found in the literature.

The separation of ethanol from its aqueous solution could be done by traditional pervaporation and MD processes. The main difference between the two processes is the role of the membrane that responds to the difference in the separation mechanisms. It is well known that the pervaporation process is based on a dense membrane and the MD is based on a hydrophobic micro-pore membrane, where the permeate flux is much more in MD than that in the pervaporation process; in turn, the higher selectivity is obtained by pervaporation. Table 3 depicts the results of the separation of ethanol from its aqueous solution at a 5 wt.% ethanol concentration using traditional pervaporation found in the literature, in order to compare against the current work results for ethanol separation by the VMD process. It is clear from Table 2 and Table 3 that there are clear differences in the permeation flux between MD and pervaporation, with the pervaporation process having an advantage in the separation factor. Therefore, due to the low permeation flux, the traditional pervaporation is more preferred to break the azeotrope composition between the ethanol and water, which is at a high ethanol concentration (95 wt.%). In the present study, the aim is separation of a low ethanol concentration (≤10 wt.%) from its solution. 

### 3.5. Taguchi Results

#### 3.5.1. The Statistical Analysis

**Figure 11:**
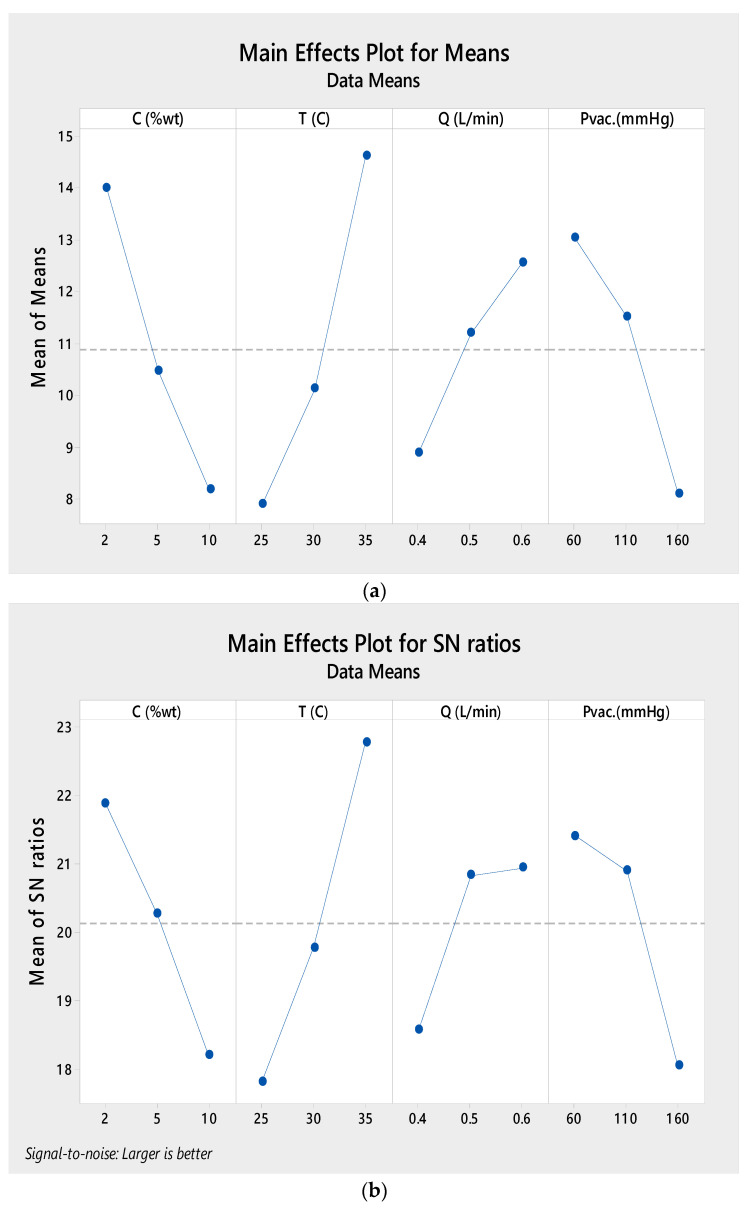
(**a**) VMD main-effect plot for the mean permeate flux. (**b**) VMD main-effect plot for the mean signal-to-noise ratio.

When the experiments were finished, the current results were transformed into S/N ratio values. The final Taguchi L_9_-OA with response values and their corresponding S/N ratio values for water vapor flow is shown in Table 4. Figure 11 provide significant impact plots for the system flux and signal-to-noise ratio, based on average values from each experimental run, respectively. These graphs are used to analyze the effects of each operational variable. As seen in Figure 11a, the flow increases as the input temperature increases due to the exponential relationship between temperature and water vapor pressure. The permeate flux increases as the feed flow rate increases. The flux decreases when the absolute pressure (vacuum zone) and ethanol concentration both rise. The parameters of the statistical analysis and descriptive statistics are shown in Table 5.

Based on the measurement of the S/N ratio, the highest performance of the VMD process was achieved at 35 °C and corresponds to a permeation flux of 21.5431 kg/m^2^·h, as shown in Table 6. Figure 11a depicts the influence of each parameter on permeate flux. On the permeate side, it can be seen that the permeate flux rises with a rising feed temperature, feed flow rate, and feed concentration but decreases with an increasing feed concentration and absolute pressure. Figure 11b shows that the S/N ratio rises as the feed temperature rises, with level 3 (35 °C) being the optimal feed temperature. This ratio increases as the feed flow rate increases, so the optimum feed flow rate is level 3 (0.6 L/min); it decreases as the concentration increases, so the optimum concentration is level 1 (2 wt.%); and it increases as the absolute pressure decreases, so the optimum absolute pressure is level 3 (60 mmHg (abs)).

#### 3.5.2. Predicted Model

**Figure 12:**
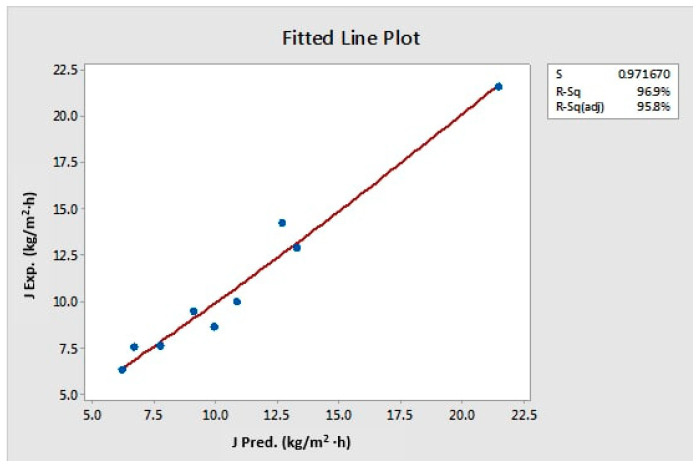
Predicted versus experimental values of the permeate.

A Minitab model for the prediction of permeate flux was created by a nonlinear regression function (Minitab version 18). The predicted model for the present VMD system based on all experimental data gives the following relation:

(8) $$J=31.3-0.700C-2.06T+18.19Q-0.0492P_{\mathrm{vac}.}+0.0456T^{2}$$

where, J is the permeate flux, which represents the dependent variable, while the feed temperature (T in °C), the vacuum pressure ($P_{\mathrm{vac}.}$ in mmHg), the feed flow rate (Q in (L/min) and the feed initial concentration (C in wt.%), respectively, represents the independent variables. The predicted and measured permeate fluxes are shown in Table 4 and Figure 12.

## 4. Conclusions

The performance test of VMD using commercial polytetrafluoroethylene (PTFE) flat-sheet membrane was carried out in the current work. It was found that the permeation flux increased as the feed temperature and flow rate increased, whereas it decreased as the ethanol concentration and absolute permeate pressure increased (in the vacuum zone). While the separation factor increased with an increasing feed temperature, feed flow rate, and absolute permeate pressure, it decreased when increasing the ethanol concentration in the feed solution. The permeation flux reached 24.145 kg/m^2^·h for a solution of 2 wt.% at a 30 °C feed temperature, 0.6 L/min feed flow rate, and 60 mmHg (abs) of absolute pressure. The permeate flux declined by about 45% with an increasing ethanol concentration from 0 to 10 wt.% at 30 °C, a 0.6 L/min feed flow rate, and 60 mmHg (abs) absolute pressure. Hence, one of the most significant advantages of the VMD process for volatile organic compounds is the minimum effect of ethanol concentration on the performance of the system. The permeate flux measured in the current study is greater than that measured in other earlier works found in the literature. It was found that the feed temperature was the most effective factor in the permeation flux, whereas the feed flow rate was the least effective component. An empirical correlation also was established for permeation flux (J) as a function of the operating variables, feed temperature (T), absolute pressure in the vacuum zone ($P_{\mathrm{vac}.}$), feed flow rate (Q), and feed concentration (C). It is worth concluding that when an aqueous ethanol solution was used, PTFE membrane failure was likely due to an increase in the chance of membrane wetting at high temperatures (above 40 °C), even at a lower initial ethanol concentration of 2 wt.%. Thus, the possibility of membrane wetting increases with an increasing temperature and ethanol concentration and decreasing absolute permeate pressure.

## Figures and Tables

**Table 1:** Characteristics of the commercial PTFE flat-sheet membrane.

Parameter	Present Work	Previous Work [8,15,16]
Membrane type/Code	PTFE/M1	PTFE/M2
Average pore size (µm)	0.2	0.22
Thickness (µm)	35	96
Porosity Ɛ%	71	78
Contact angle (°)	118.02	114
Effected membrane dimensions (cm)	4.1 × 3.1	4.1 × 3.1
LEP (bar)	5.5	unkown

**Table 2:** Comparison study on the operating conditions and permeate flux obtained by the current work with several studies presented in the literature by using VMD in various types of membranes.

Ref.	System	Mem.-Type	Characteristic of Membrane				Operating Conditions				J (kg/m^2^·h)	A
			δ (µm)	A (m^2^)	ε (%)	P.S. (µm)	Q (L/min)	Concentration	T_f_ (°C)	P_vac_ (mbar(abs))		
[27]	E-W	PTFE-FS	80	0.02	78	0.2	ø	5–10 wt.%	30	5	5.7	5.4
[28]	E-W	PTFE-FS	60	ø	60	0.2	0.627–4.764	0–5 wt.%	35–60	33.3–53.3	6.9–19.9	5.3–8.8
[29]	E-W	PP-HF	25	3 × 10^−3^	35	0.4	ø	5 wt.%	35	26–60	0.72–4.68	0.24
[18]	A-E	PTFE-FS	60	ø	60	0.2	0.05–3	2–10 wt.% A-E, IPA-W	25–35	10–80	3.6–28.8	ø
	IPA-W									10–70	1.5–25.2	
[10]	E-W	PVDF-FS	120	3.705 × 10^−3^	75	0.2	1.9–7.4	0.15–0.25 wt.%	20–45	1–49	17.1	5.2–7.9
[30]	E-W	PP-HF	1550	0.0389	75	0.2	0.06–1.31	0.15–3% *v*/*v*	23–32	28–40.4	0.36–0.5	4.4
[4]	E-W	PP-HF PVDF-FS PP-FS	25–120	0.003–9.8 × 10^−4^	35–79	0.02	ø	0.25–5 wt.%	25–70	20–60	ø	ø
[24]	E-W	PTFE-FS	50	0.002	60–70	0.22	0.83	0–5 wt.%	45–60	73–123	5–24	6.5–9
[31]	E-W	PP-Ca	ø	0.1	ø	0.2	6–25	0.5–5 wt.%	20–50	25–50	3.6	4.5–5.5
[7]	E-W	PTFE-FS	220–265	9.08 × 10^−4^	72–76	0.2–5	0.16–2.27	0.5–10 wt.%	30–50	111.4–304	2.17–44.7	1.7–2.3
[32]	E-W	PP-Ca	50–450	0.087	73	0.22	ø	5.2–12.3% *v*/*v*	26.7–47	62–114	0.6–3.2	0.7–7.4
				0.067					30.4–40.7			
[33]	E-W glucose	PTFE-Ca	220	0.0266	80	0.2	ø	175 g/L glucose 86.8 g/L Ethanol	23	ø	0.51	2.9
[9]	E-W glucose	PTFE-FS	65	0.024	72	0.5	0–2.6	5–35 g/L Ethanol 0–150 glucose	20–50	ø	1–11.5	3.5–6.6
Present work	E-W	PTFE-FS	21–51	1.269 × 10^−3^	71	0.2	0.3–0.6	0–10 wt.%	20–35	79.9–279.9	24.145	8.63

P.S.: pore size; ø: unknown; E-W: ethanol–water; A-W: acetone–water; FS: flat sheet; HF: hollow fiber.

**Table 3:** Comparison study permeate flux and separation factor obtained from several previous studies using the pervaporation process for ethanol–water separation.

Ref.	Mem.-Type	A (m^2^)	P_vac_ (mbar(abs))	T_f_ (°C)	J (kg/m^2^·h)	A
[27]	PDMS	0.02	5	30	0.06	8.1
[34]	PDMS	0.17	27	30	0.39	6.3
[35]	PDMS	0.1	5	35	0.37	4.1
[36]	PTMSP	0.0055	4	25	0.15	9.9
[37]	PDMS with zeolites	0.0028	0.3	50	0.13	13.4
[38]	PDMS	0.22	5	34	0.15	10.3
[39]	MFI with silicalite	0.00052	3	25	0.02	10
[40]	PDMS	0.22	5	35	0.09	8.2
[41]	PDMS	0.01	13	40	0.25	10
[42]	PDMS	0.017	1	65	1.6	7.8
[43]	PDMS in PA support	0.08	50	28	0.39	6.4
[44]	PDMS	0.002	3	30	0.55	8.4
[45]	POMS	0.017	1	43	0.52	9.1
[46]	PDMS	0.00088	1	41	0.17	5.5
[47]	PDMS	0.00224	10	40	0.5	8.3
[48]	PDMS/PEI	0.9	7	40	0.23	6.8
[49]	PDMS	0.04	7	27	0.004	10.6
[50]	PDMS with silicalite	0.00159	1	40	0.1	14.9
[51]	PDMS	0.16	46	35	0.4	8.5
[52]	PDMS/PEI	0.00196	40	35	0.21	7.2
[53]	PHepMS	1.385 × 10^−3^	~0.05	30	0.0028	19.9
	POMS				0.0026	18.8
	PDMS				0.0055	31
[54]	PDMS	2.55 × 10^−3^	ø	40–80	5.4	10.2

PTMSP: poly((1-trimethylsilyl)-l-propyne); POMS: polyoctylmethyl siloxane; PA: polyamide; PEI: polyethyleneimine; PDMS: polydimethylsiloxane; PHepMS: polymethylhydrosiloxane; ø: unknown.

**Table 4:** The Taguchi L_9_(3^4^) orthogonal array (OA), and the results of the experiments.

Run	Operating Parameters				Flux (Exp.) (kg/m^2^·h)	Flux (Pred.) (kg/m^2^·h)	S/N	Mean
	C (wt.%)	T (°C)	Q (L/min)	$P_{v}$ (mmHg (abs))				
1	2	25	0.4	160	6.2802	6.1967	15.9594	6.2802
2	2	30	0.5	110	14.1844	12.6885	23.0362	14.1844
3	2	35	0.6	60	21.5431	21.4596	26.6661	21.5431
4	5	25	0.5	60	9.9593	10.8394	19.9645	9.9593
5	5	30	0.6	160	8.5892	9.9468	18.6790	8.5892
6	5	35	0.4	110	12.8383	13.2610	22.1701	12.8383
7	10	25	0.6	110	7.4951	6.6984	17.4955	7.4951
8	10	30	0.4	60	7.5951	7.7333	17.6106	7.5951
9	10	35	0.5	160	9.4593	9.1200	19.5171	9.4593

**Table 5:** Parameters of the statistical analysis and descriptive statistics.

Factor	DOF	SS	Variance	F	P
Concentration (wt.%)	2	51.593	25.796	3.229	28.667%
Temperature (°C)	2	69.97	34.986	4.379	38.879%
Flow rate (L/min)	2	20.31	10.153	1.270	11.284%
Absolute pressure (mmHg (abs))	2	38.10	19.050	2.384	21.170%
Error	9	71.901	7.989		
Total	17	179.98			
descriptive statistics					
**Sample**	**N**	**Mean**	**StDev**	**SE Mean**	**95% CI**
Concentration (wt.%)	9	5.67	3.50	1.17	(2.98, 8.36)
Temperature (°C)	9	30.00	4.33	1.44	(26.67, 33.33)
Flow rate (L/min)	9	0.5000	0.0866	0.0289	(0.4334, 0.5666)
Absolute pressure (mmHg (abs))	9	110.0	43.3	14.4	(76.7, 143.3)
Flux (kg/m^2^·h)	9	10.88	4.74	1.58	(7.24, 14.53)
Separation factor	9	4.979	1.241	0.414	(4.025, 5.933)

**Table 6:** Mean flux and S/N values at all levels of the operating variables obtained from the Taguchi method.

Parameters	Level	Mean Flux (kg/m^2^·h)	S/N
Temperature (°C)	25	7.912	17.58
	30	10.123	19.78
	35	14.614	22.78
Absolute pressure (mmHg (abs))	60	13.033	21.41
	110	11.506	20.90
	160	8.110	18.05
Feed flow rate (L/min)	0.4	8.905	18.58
	0.5	11.201	20.84
	0.6	12.542	20.95
Concentration (wt.%)	2	14.003	21.89
	5	10.462	20.27
	10	8.183	18.21

## Data Availability

Not applicable.

## Nomenclature

A	membrane effective area (m^2^)
C	concentration (wt.%)
DOF	degree of freedom
J	permeate flux (kg/m^2^·h)
K	over all mass transfer coefficient
$k_{f}$	mass transfer coefficient of feed layer
$k_{m}$	mass transfer coefficient of membrane layer
$k_{p}$	mass transfer coefficient of feed layer
L	effective length of membrane (m)
$P_{mf}$	vapor pressure of the solution at the membrane feed side
$P_{mp}$	vapor pressure of the solution at the membrane permeate (vacuum) side
P°	the vapor pressure for pure water
P	number of parameters
Q	the rate of feed flow in (L/min)
R^2^	coefficient of determination
R	coefficient of correlation
S/N	signal to noise ratio
$T_{f}$	feed temperature (°C)
$T_{mf}$	feed temperature at the membrane surface (°C)
t	operational time (hour)
V	volume of fresh water (L)
W	effective width of membrane (m)
$x_{e,f}$	the mole fractions of ethanol in the bulk liquid feed
$x_{w,f}$	the mole fractions of water in the bulk liquid feed
$y_{e,p}$	the mole fractions of ethanol in the permeate
$y_{w,p}$	the mole fractions of water in the permeate
Greek Symbols	
δ	membrane thickness
Ɛ	porosity
ρ	density (kg/L)
α	separation factor
